# Supplementary material for: RhizoVision Explorer: open-source software for root image analysis and measurement standardization
Source: AoB Plants. 2021 Sep 13;13(6):plab056. doi: 10.1093/aobpla/plab056 (PMC8598384; doi:10.1093/aobpla/plab056)
Supplement: plab056_suppl_Supplementary_Materials [file plab056_suppl_supplementary_materials.pdf]

Supplemental Table 1. Combinations of 12 wires used for the copper wire image data set, ranging from 1 to 12 wires in a scan. For each of 6 wire gauges (diameters), two wires of approximately 30 cm length were used and denoted as wire 1 or 2. File names for the scans indicate combinations of the gauges and wire numbers used, and the number (#) of wires in a scan are provided.

AWG Gauge	File Name	#
40	G_40_W1_600_dpi.tif	1
40	G_40_W2_600_dpi.tif	1
32	G_32_W1_600_dpi.tif	1
32	G_32_W2_600_dpi.tif	1
28	G_28_W1_600_dpi.tif	1
28	G_28_W2_600_dpi.tif	1
22	G_22_W1_600_dpi.tif	1
22	G_22_W2_600_dpi.tif	1
16	G_16_W1_600_dpi.tif	1
16	G_16_W2_600_dpi.tif	1
10	G_10_W1_600_dpi.tif	1
10	G_30_W2_600_dpi.tif	1
40	MD_40_40_W12_600_dpi.tif	2
40, 32	Mixed_40_32_W1_600_dpi.tif	2
40, 32	Mixed_40_32_W2_600_dpi.tif	2
40, 32	MD_40_32_40_32_W1122_600_dpi.tif	4
40, 32, 28	Mixed_40_32_28_W1_600_dpi.tif	3
40, 32, 28	Mixed_40_32_28_W2_600_dpi.tif	3
40, 32, 28	MD_40_32_28_40_32_28_W111222_600_dpi.tif	6
40, 32, 28, 22	Mixed_40_32_28_22_W1_600_dpi.tif	4
40, 32, 28, 22	Mixed_40_32_28_22_W2_600_dpi.tif	4
40, 32, 28, 22	MD_40_32_28_22_40_32_28_22_W11112222_600_dpi.tif	8
40, 32, 28, 22, 16	Mixed_40_32_28_22_16_W1_600_dpi.tif	5
40, 32, 28, 22, 16	Mixed_40_32_28_22_16_W2_600_dpi.tif	5
40, 32, 28, 22, 16	MD_40_32_28_22_16_40_32_28_22_16_W1111122222_600_dpi.tif	10
40, 32, 28, 22, 16, 10	Mixed_40_32_28_22_16_10_W1_600_dpi.tif	6
40, 32, 28, 22, 16, 10	Mixed_40_32_28_22_16_10_W2_600_dpi.tif	6
40, 32, 28, 22, 16, 10	MD_40_32_28_22_16_10_40_32_28_22_16_10_W111111222222_600_dpi.tif	12
